# Thrombotic risk assessment in antiphospholipid syndrome: the role of new antibody specificities and thrombin generation assay

**DOI:** 10.1186/s12948-016-0043-2

**Published:** 2016-07-15

**Authors:** Savino Sciascia, Simone Baldovino, Karen Schreiber, Laura Solfietti, Massimo Radin, Maria J. Cuadrado, Elisa Menegatti, Doruk Erkan, Dario Roccatello

**Affiliations:** Department of Clinical and Biological Sciences, Center of Research of Immunopathology and Rare Diseases- Coordinating Center of the Network for Rare Diseases of Piedmont and Aosta Valley, San Giovanni Hospital, University of Turin, piazza del donatore di Sangue 3, 10054 Turin, Italy; Guy’s and St Thomas’ NHS Foundation Trust, London, UK; Department of Rheumatology, Copenhagen University Hospital, Copenhagen, Denmark; Louise Coote Lupus Unit, Guy’s and St Thomas’ NHS Foundation Trust, London, UK; Barbara Volcker Center for Women and Rheumatic Diseases, New York, USA; Hospital for Special Surgery, New York, USA; Department of Clinical and Biological Sciences, SCDU Nephrology and Dialysis, S. Giovanni Bosco Hospital, University of Turin, Turin, Italy

**Keywords:** Antiphospholipid syndrome, Antiphospholipid antibodies, Thrombosis, Pregnancy loss, Miscarriages, Anti-β2-glycoprotein-I antibodies, Thrombin generation assay, Lupus anticoagulant, Clinical trials, Anti-prothrombin, APS action, Risk assessment, Antiphospholipid antibody

## Abstract

Antiphospholipid syndrome (APS) is an autoimmune condition characterized by the presence of antiphospholipid antibodies (aPL) in subjects presenting with thrombosis and/or pregnancy loss. The currently used classification criteria were updated in the international consensus held in Sidney in 2005. Vascular events seem to result of local procoagulative alterations upon triggers influence (the so called “second-hit theory”), while placental thrombosis and complement activation seem to lead to pregnancy morbidity. The laboratory tests suggested by the current classification criteria include lupus anticoagulant, a functional coagulation assay, and anticardiolipin and anti-β2-glycoprotein-I antibodies, generally detected by solid phase enzyme-linked immunosorbent assay. The real challenge for treating physicians is understanding what is the actual weight of aPL in provoking clinical manifestations in each case. As thrombosis has a multi-factorial cause, each patient needs a risk-stratified approach. In this review we discuss the role of thrombotic risk assessment in primary and secondary prevention of venous and arterial thromboembolic disease in patients with APS, focusing on new antibody specificities, available risk scoring models and new coagulation assays.

## Background

The strive for personalised medicine can be traced in its origin to Hippocrates’ times: the assessment of the four humours—blood, phlegm, yellow bile and black bile—were essential to determine the correct treatment for each individual patient. Nowadays, the emphasis on disease prediction and prevention remains the main hallmark and challenge of individualised and personalised medicine and is largely dependent on advances in research and published literature. One of the most ubiquitous examples to personalise medicine is the development of risk stratification or scoring models in order to predict the development of any given clinical outcome or disease.

The eagerness to develop and validate reliable scoring models for the prediction of clinical outcomes in order to improve individual clinical care has motivated researchers within the field of autoimmune diseases to propose useful scoring models [1–4]. Especially in areas with a variety of clinical effectors and outcome variables, solid scoring systems are essential to provide a valuable guidance in clinical practice to advise clinicians to the right treatment strategy.

The antiphospholipid syndrome (APS) is a systemic autoimmune disease, which is defined by the presence of thromboses and/or obstetric morbidity in patients persistently positive for antiphospholipid antibodies (aPL). The classification criteria for APS have been outlined in the original Sapporo criteria, and have more recently been updated in the Sydney criteria [5] (Table 1).

**Table 1:** APS classification criteria, modified from Miyakis et al. [5]

Vascular thrombosis:	≥1 Clinical episode of arterial, venous or small vessel thrombosis. Thrombosis must be objectively confirmed. For histopathological confirmation, thrombosis must be present without inflammation of the vessel wall
Pregnancy morbidity:	1. ≥1 Unexplained death of a morphologically normal fetus ≥10 weeks of gestation 2. ≥1 Premature delivery of a morphologically normal fetus <34 weeks gestation because of: Severe pre-eclampsia or eclampsia defined according to standard definition Recognised features of placental insufficiency 3. ≥3 Unexplained consecutive miscarriages <10 weeks gestation, with maternal and paternal factors (anatomic, hormonal or chromosomal abnormalities) excluded
Laboratory criteria:	The presence of antiphospholipid antibodies (aPL), on two or more occasions at least 12 weeks apart and no more than 5 years prior to clinical manifestations, as demonstrated by ≥1 of the following Presence of lupus anticoagulant in plasma Medium to high-titre anticardiolipin antibodies (>40 GPL or MPL, or >99th ‰) of IgG or IgM isoforms Anti-β_2_ glycoprotein-I antibody (anti-β_2_GPI) of IgG or IgM present in plasma

aPL comprehends a heterogeneous group of circulating immunoglobulins including lupus anticoagulant (LA), anticardiolipin antibodies (aCL) and anti-β2glycoprotein-I (anti-β2GPI). Their persistent presence is related to diverse clinical phenomena including arterial and venous thrombosis, pregnancy complications and other common clinical manifestations such as livedo reticularis or thrombocytopenia. As the presence of aPL does not always lead to thrombotic events in every individual with aPL, aPL are necessary, but not sufficient for clinical manifestations such as thromboembolic events or pregnancy morbidity. A “second hit theory” has been proposed, suggesting that other factors may trigger a clinical manifestation in individuals with aPL [6, 7].

Shi et al. studied the prevalence of LA and aCL in 499 healthy Australian blood donors and found LA to have a prevalence of 3.6 % and aCL, 4.6 % [8], whereas 1 % of healthy control patients in the Leiden Thrombophilia study were found to have LA and 3.6 %, anti- β2GPI antibodies [9]. The prevalence of aPL is higher in patients with other autoimmune conditions such as rheumatoid arthritis or systemic lupus erythematosus (SLE), where up to 40 % are persistently positive for aPL [10, 11]. Given their heterogeneity, their combinations and titres researchers have focused on exploring their link to different risks of clinical outcomes [12–15]. Furthermore, scoring models for the use in aPL positive individuals and patients with APS to predict the risk of thrombosis have been developed and validated by different groups [1, 2, 16, 17].

In this review we discuss the role of thrombotic risk assessment for venous and arterial thromboembolic disease in aPL-positive patients, focusing on new antibodies specificities, available risk scoring models and new coagulation assays. A comprehensive analysis of the therapeutic options for the management of aPL-positive patients is beyond the scope of this review; detailed information about current and potential future management strategies can be found elsewhere [6].

## Clinical manifestations of APS

APS is widely considered as the major acquired thrombophilia, which can affect any vascular bed (arterial, venous and the microvasculature). This may explain the variety of clinical manifestations described in APS patients. Albeit most of the clinical manifestations can be attributed to underlying thrombosis, inflammation, complement, platelet activation and macrophages have been shown to play crucial roles in the pathophysiology of the syndrome [6].

In a European cohort of 1000 APS patients, deep vein thrombosis (DVT) and pulmonary embolism (PE) were the most frequent clinical manifestations of the syndrome, whereas the most frequent arterial manifestations are neurological, such as stroke or transient ischemic attacks (TIA) [18]. Other neurological features include migraine headaches, memory loss and epilepsy. Thrombocytopenia and livedo reticularis are the most important haematological and dermatological characteristics, respectively, and can be found in up to 20 % of APS patients [18].

Pregnancy morbidity includes unexplained fetal death, premature birth before 34 weeks of gestation due to severe pre-eclampsia, eclampsia or placental insufficiency or recurrent first trimester miscarriage. Pre-eclampsia, premature birth or fetal loss are the most common manifestations and occur in 10–20 % of APS pregnancies [18].

## Laboratory testing for aPL

aPL can be detected with three tests, all of which should be performed in any individual patient before the presence of aPL can be excluded or confirmed. The assays comprise test for the presence of lupus anticoagulant, anticardiolipin antibodies and anti-β2GPI [19, 20]. Individuals may be positive for one, two or three of these tests and are in the literature referred to as single, double or triple positive, respectively.

### Lupus anticoagulant testing

LA is a functional assay measuring the ability of aPL to prolong phospholipid—dependent clotting assays. LA testing has been difficult to standardise, and no single test appears to be adequate, indicating the heterogeneity of different individuals aPL. As no coagulation test has 100 % sensitivity, the 2009 Scientific and Standardisation Committee of the International Society on Thrombosis and haemostasis guidelines recommend two assays of different assay principle, the diluted Russell viper venom (dRVVT) test and a sensitive activated partial thromboplastin time (aPTT), with silica as an activator because of its sensitivity for LA [19]. According to the updated guidelines, the laboratory detection of LA should be based on the following criteria: (1) prolongation of phospholipid dependent clotting test, in particular when the phospholipid content of test system is low; (2) lack of correction of the prolonged clotting time by addition of a small amount of normal plasma (thereby to exclude factors deficiency); (3) correction by the presence of high concentration of phospholipid such as the use of platelet fragments, which will remove all the aPL, or by the use of a reagent that is poorly responsive for LA effect [19]. Similarly, current guidelines for LA detection recommend mixing test interpretation with either a mixing test-specific cut-off (MTC) or index of circulating anticoagulant (ICA) [19]. Very recently, Moore et al. [21] retrospectively applied MTC and ICA assessment to raw data of 350 LA positive plasmas from non-anticoagulated patients to compare detection rates of inhibition. They concluded that it is valuable to maximise mixing test interpretation as the dilution can lead to false negative results. Consequently, MTC is superior to ICA in detecting the in vitro inhibition of LA and might be a useful tool when assessing the risk in patients suspected for APS.

### Anticardiolipin antibodies

aCL are usually detected by either radioimmunoassay or ELISA, using cardiolipin as the solid phase antigen. Serum is used for the aCL assays. IgG, IgM and/or IgA isotypes concentrations are expressed as GPL, MPL and/or IgA units, respectively, where 1 unit represents the binding activity of 1 mg/ml of affinity purified aCL antibody. aCL IgG and IgM are currently part of the APS classification criteria [5], whereas the clinical value of aCL IgA remains subject of discussion as outlined below.

In general, positive LA tests are more specific for the APS, whereas aCL are more sensitive [9, 17]. The specificity of aCL for APS increases with the titre and is higher for the IgG than for the IgM isotype. However, some patients may have only a positive IgM test, and a few are only IgA positive.

## Anti-β2glycoprotein-I and anti-domain I-β2glycoprotein-I antibodies

The development of anti-β2GPI immunoassays followed the observation that many aCL are directed to an epitope on β2-glycoprotein. However, in patients with clinical features of APS, anti-β2GPI antibodies are rarely the sole antibodies detected [15].

The β2GPI has five homologous domains (D1 to D5) and recently several studies focused their attention on the epitope distribution of anti-β2GPI antibodies, in order to identify the pathogenic specificities [22]. The main epitope that has been found to be associated with APS involves regions of D1 [23]. Recent promising data support the association between anti-β2GPI-D1 and APS clinical manifestation. Recent studies have found that patients with multiple aPL have a higher prevalence and higher titers of anti-β2GPI-D1 antibodies [22, 23]. International multi-centre evaluations have recently confirmed the preliminary reports supporting the associations between an history of thrombosis (mostly venous) and anti-β2GPI-D1 antibodies (reviewed in [24]). Taken the above together, anti-β2GPI-D1 antibodies might be a promising biomarker for risk assessment in APS. Further studies are warranted to validate this hypothesis.

### IgA aPL isotypes

To date, no conclusive prospective study has offered a clear view on the usefulness of IgA aPL antibodies testing. IgA aPL antibodies have a low prevalence and in most cases they are found along with other aPL, but they have failed to enhance the diagnostic accuracy when routinely tested [25].

To date, the use of IgA isotypes for both aCL and anti-β2GPI are not a part of the routine diagnostic algorithm [20]. However some data suggested a role of isolated positivity for IgA anti-β2GPI with clinical APS symptoms might help to identify additional patients, recommending this tests when other aPL are negative. Based on the present evidence IgA aPL testing should be considered for thrombotic assessment risk only in selected patients, in particular when other aPL tests are negative, in the presence of clinical APS signs and/or symptoms, mainly associated with SLE [20].

### Non criteria aPL

#### Antiprothrombin antibodies

The role of aPL assays for the detection of autoantibodies to phospholipids other than criteria antiphospholipid antibodies (for example anti-prothrombin or anti-phosphatidylserine) is an ongoing subject of debate. These antibodies are directed to negatively charged phospholipids other then cardiolipin. Specifically, antibodies to prothrombin can be detected by directly coating prothrombin on irradiated ELISA plates (aPT) or by using the phosphatidylserine/prothrombin complex as antigen (aPS/PT) [26]. Although these antibodies can co-exist in the same patient, they are two distinct populations of antibodies. In fact, aPS/PT (rather than aPT) has been shown to help to establish the diagnosis of APS and the associated risk for both arterial and/or venous thrombosis or pregnancy morbidity [27]. Their clinical importance is far from being fully explored, but the presence of these other aPL antibodies might represent a new tool for risk stratification, especially for those patients negative for the classic aPL.

#### Anti-phosphatidylethanolamine antibody

In solid assays, sera from patients with APS usually react to negatively charged phospholipids (PL) and PL cofactors such as β2-glycoprotein I (β2GPI). Binding to non negative charged PL (such as neutral PL, e.g. phosphatidylethanolamine) is less frequently observed [28].

Phosphatidylethanolamine (PE) is one of the main lipid components of the cell membranes, being mostly located in the inner leaflet [28]. In 1989 Staub et al. [28] reported the first case of primary APS whereby a LA was accompanied not by an aCL, but by an antibody to PE (aPE).

The clinical significance of aPE in patients with the thrombosis and/or pregnancy morbidity but negative for criteria aPL (so-called seronegative APS) is a very hot topic under investigation. Hirmerova et al. [29] showed that in 140 patients with venous thromboembolism, of non-criteria aPL antibodies, only aPE was significantly more prevalent compared to controls, with minor overlapping with the criteria aPL. Moreover, aPE antibodies were associated with a higher risk of thrombosis in a multicentre study including 270 patients with thrombotic disease and 236 matched controls [30]. Of note, more than 60 % of 40 aPE-positive patients were negative for the APS laboratory criteria. Similar results were found by the same group [31]. The screening for IgG, IgM and IgA aPE, seems to increase the diagnostic yield in APS [32], particularly when traditional laboratory criteria for APS are lacking [33]. After more than 25 years from the first description of aPE antibodies in a patient with primary APS [34], the clinical value of these antibodies in individuals with unexplained thrombosis and pregnancy morbidity remains intriguing. Whilst solid evidence supporting the inclusion of aPE as classification criteria for APS is lacking, one may consider testing for aPE in patients with clinical features suggestive of APS without the presence of criteria aPL [35].

#### Anti-vimentin antibodies

Vimentin is a ubiquitous protein part of the cytoskeleton intermediate filament structure. Anti-vimentin antibodies were first described in patients with SLE, and they reportedly exhibit significant association with the presence of aCL [36]. Albeit anti-vimentin antibodies have been shown to activate platelets and leukocytes with increased expression of P-selectin, fibrinogen, TF, and platelet-leukocyte conjugates [37], the diagnostic role of in the context of APS is still largely undefined. Ortona et al. [38] demonstrated that vimentin is capable of binding cardiolipin in vitro, possibly as a result of electrostatic interaction between its positively charged amino acids and the negatively charged amino acids of cardiolipin. The antivimentin/cardiolipin antibody complexes were found in a large proportion of patients with clinical features suggestive of APS without criteria aPL patients tested and almost all those with APS [38]. These findings led the authors to consider vimentin as a new antigenic cofactor for aPL in APS and the vimentin/cardiolipin complex as a molecular target of the antibodies in APS patients. It is important to note, however, that antivimentin/cardiolipin antibodies also have been detected in SLE and RA patients, so despite their high sensitivity, these antibodies are not very specific for APS.

#### Anti- annexin A5 and anti-annexin 2 antibodies

Anti-AnxA5 antibodies (aAnxA5) have also been described in APS. Despite promising results observed in mice where aAnxA5 have been associated with placental thrombosis and fetal absorption [39], conflicting findings have been observed concerning the association of aAnxA5 with a history of pregnancy-related morbidity in humans [40–42]. Similarly, de Laat et al. [43] did not observe any association between aAnxA5 and a history of thrombosis.

Annexin 2 is a cofactor for plasmin generation and cell-surface localization of fibrinolytic activity, (mainly monocytes, placental syncytiotrophoblasts and endothelialium) [44, 45]. Cesarman-Maus et al. were the first to describe the high prevalence of anti-annexin 2 antibodies in patients with APS [46]. Several observations might be in line with a pathogenic role for anti-annexin 2 antibodies in APS (e.g. induction of TF expression on endothelial cells; second, prevention of placental annexin 2 from acting as a cofactor during plasmin generation) [39]. However, their clinical relevance remains a matter of debate and needs to be confirmed with experimental data as well as longitudinal studies involving sufficient number of patients.

## Thrombin generation assay and APS

A significant number of studies highlight that an altered thrombin generation may lead to pathologic processes, meanly haemorrhagic or thrombotic diseases [47]. Recent tests based on the continuous detection of thrombin generation under in vitro conditions that mimic more closely what occurs in vivo, has motivated a reinvestigation of the balance between pro-coagulants and anti-coagulants in patients with various haemostatic disorders. Furthermore, the evaluation of an individual’s thrombin-generation potential serves as a useful estimator of the total coagulation potential, i.e. hyper- or hypocoagulability, when compared to traditional coagulation tests [48]. The clinical utility of thrombin generation tests however remains subject to highly specialised teams and is far from being used in standard clinical practice. However, TGA (thrombin generation assay) is a simple and reproducible technique that potentially can be used in coagulation laboratories. The test measures the amount of thrombin formed immediately after citrated plasma recalcification by adding exogenous activators (human recombinant tissue factors (TF) and phospholipids). Concentration of generated thrombin in the sample is recorded and then calculated from the fluorescence over time variation of the substrate, resulting in a thrombin generation curve. The following parameters are acquired: Lag time (tLag, time until thrombin burst), time to Peak thrombin generation (tPeak), peak amount of thrombin generation (Peak), velocity of thrombin generation (vel), and the total amount of thrombin generated (AUC, Area Under the thrombin generation Curve) [49].

The clinical utility of TGAs in assessing the thrombotic risk has been a matter of growing interest [47]. However, to date, TGA has been applied only for research purpose and its role in clinical practice still needs to be evaluated.

Few studies [50–53] described an association between an unbalance in TGA parameters and thrombotic risk in APS. Dienava-Verdoold et al. [50] showed successful cloning of patient-derived mAbs that require domain I of β2GPI for binding, and that display LA activity that is dependent on their affinity for β2GPI. When assessing the thrombin generation by using calibrated automated thrombography, it has been shown that lag time is influenced by the presence of anti-β2GPI antibodies, and that the prolongation of the lag time was similar to the clotting time prolongation observed in the dRVVT and the aPTT assay.

Regnault et al. applied the thrombinography assessed by the conversion of a fluorogenic substrate in order to investigate the presence of acquired resistance to activated protein C (APC) in patients with LA [51]. They observed the complete process of thrombin formation and decay and its delimitation by the protein C system in eight consecutive LA-patients (all patients were not taking anticoagulation therapy). In 7 out of 8 patients they observed a long lag-time before the thrombin burst (LA effect) together with a marked inability of APC to diminish the thrombin activity. The lag-phase was however prolonged to some degree by APC when compared to controls. The effects were more evident in the presence of phospholipids from patients’ platelets than with added phospholipids. Thus, thrombinography demonstrates APC resistance in LA-patients despite the occurrence of long lag-times (clotting times).

These observations were confirmed by Zuily et al. [53] when they studied acquired APC resistance in patients with aPL using a TGA. A parameter summarizing APC inhibition of thrombin generation with increasing APC concentrations (IC(50)-APC) was increased in all patient groups compared to controls: median values were 15.3 (interquartile range, IQR, 9.7–34.0) in patients with primary APS, 27.3 (IQR 23.5–43.5) in patients with SLE without APS, 64.1 (IQR 25.9–65.0) in patients with SLE/APS compared to 10.4 [IQR 8.5–15.8] in controls, respectively p = 0.003, p = 0.0001 and p = 0.0001.

More recently, Efthymiou et al. [52] compared the degree of anticoagulation intensity in thrombotic APS and non-APS patients (50 in each group) on long-term warfarin. The group measured the INR with two widely available thromboplastins with instrument-specific ISI values in order to investigate the potential role of amidolytic FX levels and thrombin generation. While there were no overall differences in INR between reagents or patient groups.

ETP and peak thrombin showed significant inverse correlations with the INR, suggesting that TGA testing may be helpful in the determination of true anticoagulant intensity in APS patients, including those with ≥3.5 INR. Thrombin generation testing also highlighted a subgroup of APS patients with increased peak thrombin relative to the intensity of anticoagulation as assessed by INR and FX, supporting thrombin generation testing as a possible tool for the identification of ongoing prothrombotic states in patients on warfarin.

Overall, TGA seems a promising tool to further asses the thrombotic risk in patients with aPL; besides, TG testing may be useful in identifying subgroups of patients at higher risk such as those with an ongoing prothrombotic state and apparently adequate anticoagulation intensity as assessed by INR.

## Thrombotic risk assessment in aPL carriers and APS

aPL titres as well as their single, double or triple presence, have all been suggested to have a different distinct clinical significance [12, 13, 15, 17].

In general, the presence of aPL in individuals without any clinical manifestations, i.e. aPL carriers, can generally been seen as a risk factor for first time thromboembolic events [12]. LA has been shown to be a better predictor for thrombosis compared to any other aPL. In details, in 2003 Galli et al. showed in a systematic review that LA is a strong risk factors for both arterial and venous thrombosis. The group assessed the risk of thrombosis associated with LA and aCL on studies including 753 patients and 234 controls and found a significant relation between LA and thrombosis with an OR ranging from 5.7 to 9.4 aCL in turn was never associated with arterial or venous thrombotic events [54]. Of note, when the systematic review was performed, anti-β2GPI were routinely tested and the studies were performed prior to the latest amendment of the APS classification criteria where anti-β2GPI was included into the criteria.

Conversely, De Groot et al. showed in their Leiden cohort, that the presence of LA alone without the presence of anti-β2GPI (or antiprothrombin antibodies) was not significantly associated with a risk for a first DVT (OR 1.3, 95 % CI 0.3–6.0). However, in patients who tested positive for LA and anti-β2GPI antibodies (or anti-prothrombin) the OR of a first time deep venous thrombosis increased to 10.1 (95 % CI 1.3–79.8) [9].

More recently, Pengo et al. showed that the presence of triple positive patients carries a higher risk of thrombosis (and adverse pregnancy outcome) compared to patients with positivity for only one aPL. The risk associated to the so-called ‘triple positivity’ (defined as the simultaneous positivity for LA, aCL and anti-β2GPI) was assessed in a study describing clinical outcomes of one hundred thirty-three patients after 1 year follow-up, 76 patient after 5 year follow-up and 23 patients after 10 year follow-up. Over 30 % had a thromboembolic event during their follow up. Interestingly, there were more arterial events compared to venous events (25 had a venous thromboembolic event and 30 patients experienced an arterial event). However, it is worth noting that compliance with regards to anticoagulation therapy was not specified [55].

Otomo et al. expanded on this principle and developed the aPL-score (aPL-s), with the aim to evaluate whether aPL titres influence the risk of thrombosis, comparing high to medium/low titres of aCL and anti-β2GPI IgG and IgM, respectively. The group showed that high levels of IgG aCL, anti-β2GPI (and also anti-phosphatidylserine and anti-prothrombin antibodies) were closely related to the clinical manifestations of APS. In their study the aPL-score related with a history of thrombosis or pregnancy morbidity. Moreover, the aPL-s score was shown to be of predictive value for the recurrence and/or new onset of thrombotic events [1]. These preliminary observations were independently validated [16].

Moving towards the concept of aPL as a risk factor, our group recently published a comprehensive series of studies developing and validating the global APS score (GAPSS) in different patients populations [2]. The GAPPS score combines independent risk factors for thrombosis and pregnancy loss, taking into account aPL profiles (criteria aPL and non criteria aPL), as well as conventional cardiovascular risk factors and autoimmune antibody profiles.

Among all the computed variables (extensive aPL testing, cardiovascular risk factors evaluation, autoimmune profile), multivariate logistic regression analysis showed that only arterial hypertension, hyperlipidaemia, LA, aCL IgG and/or IgM, anti-β2GPI IgG and/or IgM and aPS/PT IgG and/or IgM were independent risk factors for thrombosis and/or pregnancy morbidity. All variables were computed as dichotomized, in order make GAPSS more widely adoptable. aPL positivity was assessed according to the updated APS classification criteria [5].

The GAPSS includes IgG/IgM aCL (five points), IgG/IgM anti-β2GPI (four points), LA (four points), IgG/IgM anti-phosphatidylserine-prothrombin complex antibodies (three points), hyperlipedaemia (three points) and arterial hypertension (one point) (Table 2).

**Table 2:** The global antiphospholipid syndrome score (GAPSS)

	Factor	Point value^a^
aPL^a^	Anticardiolipin IgG/IgM	5
	Anti-β2-glycoprotein IgG/IgM	4
	Lupus anticoagulant	4
	Anti-prothrombin/phosphatidylserine complex (aPS/PT) IgG/IgM	3
Cardiovascular risk factors	Hyperlipidemia^b^	3
	Arterial hypertension^c^	1

Cardiovascular risk factors were assessed following National Institute for Health and Clinical Excellence guidelines (Excellence NIfHaC. Lipid modification: cardiovascular risk assessment and the modification of blood lipids for the primary and secondary prevention of cardiovascular disease. 2010. URL: http://www.nice.org.uk/guidance/CG181 and Excellence NIfHaC. Hypertension. 2011. URL: http://www.nice.org.uk/guidance/QS28)

^a^aPL positivity was assessed according to the updated APS classification criteria [5]

^b^Serum total and high-density lipoprotein cholesterol levels were determined with standardized enzymatic methods and interpreted according to current cutoff values (total cholesterol of <5.0 mmol/l; <3.0 mmol/l for low-density lipoprotein cholesterol) (British Cardiac Society, British Hypertension Society, Diabetes UK, HEART UK, Primary Care Cardiovascular Society, Stroke Association. JBS 2: Joint British Societies’ guidelines on prevention of cardiovascular disease in clinical practice. Heart 2005; Suppl 5:v1–52)

^c^Arterial hypertension was defined as appropriately sized high blood pressure cutoff (140/90 mm Hg or higher) at least in two occasions or use of oral antihypertensive medications

The GAPSS model was developed in patients with systemic lupus erythematosus (SLE) and higher GAPS scores were observed in patients who experienced thrombosis and/or pregnancy loss compared with those without clinical events. Moreover, the GAPSS score was evaluated in an other prospective study of 51 SLE patients [56] and in 62 consecutive patients with primary APS (PAPS) [3]. In both cohorts an increase in the GAPSS was found in patients who experienced thrombosis during the follow up period compared with those without events. Furthermore, higher GAPSS scores were observed in patients who experienced thrombosis compared with those with pregnancy morbidity alone. In more detail, patients with GAPSS values higher or equal of 11 were shown to have a higher risk of recurrences.

The GAPSS model was further applied and validated by two independent groups. Oku et al. [57] described, in a large cohort of rheumatologic patients, that APS manifestations (thrombosis or pregnancy morbidity) were experienced by patients with higher GAPSS values compared to patients without APS manifestations. Recently, in another large cohort with APS and SLE patients, Zuily et al. described mean GAPSS values significantly higher for patients who underwent a thrombotic event compared to those who didn’t experienced a thrombotic event [53].

## Conclusions and future research agenda

An individual thrombotic risk assessment and “risk stratification” are fundamental for good clinical practice when evaluating patients with persistent aPL. Currently, this assessment is mostly limited to traditional cardiovascular and venous risk factors as well as aPL (LA test, aCL, and anti-β2GPI) profile. However, the field is moving towards better “risk stratification” given that new aPL tests, biologic risk assessment, e.g., thrombin generation, and aPL-specific thrombosis risk calculators have been studied in aPL-positive patients. Thus, future research agenda is promising and requires international collaboration.

AntiPhospholipid syndrome alliance for clinical trials and InternatiOnal networking (APS ACTION) is the first-ever international research network that has been created specifically to design and conduct well-designed, large-scale, multicenter clinical studies in persistently aPL-positive patients.

Among other activities [58–61], in early 2012, APS ACTION launched an international clinical database and repository “registry” of persistently aPL-positive patients with or without systemic autoimmune diseases, which also includes annual blood collection for aPL-testing and future basic science studies. To date, the network is composed of 49 multidisciplinary physicians and investigators interested in APS research from 29 international centers (www.apsaction.org). We believe that collaborative efforts such as APS ACTION will help better define thrombosis risk assessment in APS.

## Abbreviations

APS: antiphospholipid syndrome
aPL: antiphospholipid antibodies
TGA: thrombin generation assay
LA: lupus anticoagulant
aCL: anticardiolipin antibodies
SLE: systemic lupus erythematosus
Anti-β2GPI: anti-β2-glycoprotein-I antibodies
PE: phosphatidylethanolamine
aPE: anti-phosphatidylethanolamine antibodies
GAPSS: global APS score
aPS/PT: anti-phosphatidylserine/prothrombin complex antibodies
aPT: anti-prothrombin antibodies
aAnxA5: anti-AnxA5 antibodies
APC: activated protein C
dRVVT: diluted Russell viper venom time
aPTT: activated partial thromboplastin time
PE: pulmonary embolism
DVT: deep vein thrombosis
TIA: transient ischemic attacks
MTC: mixing test-specific cut-off
ICA: index of circulating anticoagulant

## References

[1] Otomo K, Atsumi T, Amengual O, Fujieda Y, Kato M, Oku K et al. Efficacy of the antiphospholipid score for the diagnosis of antiphospholipid syndrome and its predictive value for thrombotic events. Arthritis Rheum. 2012;64:504–12. Available from http://www.ncbi.nlm.nih.gov/pubmed/21953404. 10.1002/art.33340 21953404

[2] Sciascia S, Sanna G, Murru V, Roccatello D, Khamashta MA, Bertolaccini ML. GAPSS: the global anti-phospholipid syndrome score. Rheumatology. 2013;52:1397–403. Available from http://www.ncbi.nlm.nih.gov/pubmed/23315788. 10.1093/rheumatology/kes388 23315788

[3] Sciascia S, Sanna G, Murru V, Roccatello D, Khamashta MA, Bertolaccini ML. The global anti-phospholipid syndrome score in primary APS. Rheumatology. 2015;54:134–8. Available from http://www.ncbi.nlm.nih.gov/pubmed/25122726. 10.1093/rheumatology/keu307 25122726

[4] Wang S, Hsieh E, Zhu L, Wu B, Lu L. Comparative assessment of different health utility measures in systemic lupus erythematosus. Sci Rep. 2015;5:13297. Available from http://www.ncbi.nlm.nih.gov/pubmed/26293686. 10.1038/srep13297 PMC4543990 26293686

[5] Miyakis S, Lockshin MD, Atsumi T, Branch DW, Brey RL, Cervera R, et al. International consensus statement on an update of the classification criteria for definite antiphospholipid syndrome (APS). J Thromb Haemost. 2006;4:295–306. Available from http://www.ncbi.nlm.nih.gov/pubmed/16420554. 10.1111/j.1538-7836.2006.01753.x 16420554

[6] Ruiz-Irastorza G, Crowther M, Branch W, Khamashta MA. Antiphospholipid syndrome. Lancet. 2010;376:1498–509. Available from http://www.ncbi.nlm.nih.gov/pubmed/20822807. 10.1016/S0140-6736(10)60709-X 20822807

[7] Meroni PL, Borghi MO, Raschi E, Tedesco F. Pathogenesis of antiphospholipid syndrome: understanding the antibodies. Nat Rev Rheumatol. 2011;7:330–9. Available from http://www.ncbi.nlm.nih.gov/pubmed/21556027. 10.1038/nrrheum.2011.52 21556027

[8] Shi W, Krilis SA, Chong BH, Gordon S, Chesterman CN. Prevalence of lupus anticoagulant and anticardiolipin antibodies in a healthy population. Aust NZ J Med. 1990;20:231–6. Available from http://www.ncbi.nlm.nih.gov/pubmed/2115326. 10.1111/j.1445-5994.1990.tb01025.x 2115326

[9] de Groot PG, Lutters B, Derksen RHWM, Lisman T, Meijers JCM, Rosendaal FR. Lupus anticoagulants and the risk of a first episode of deep venous thrombosis. J Thromb Haemost. 2005;3:1993–7. Available from http://www.ncbi.nlm.nih.gov/pubmed/16102105. 10.1111/j.1538-7836.2005.01485.x 16102105

[10] Jeleniewicz R, Majdan M, Targońska-Stępniak B, Dryglewska M. Prevalence of antiphospholipid antibodies in rheumatoid arthritis patients and relationship with disease activity. Pol Arch Med. 2012;122:480–6. Available from http://www.ncbi.nlm.nih.gov/pubmed/22910193. 10.20452/pamw.1415 22910193

[11] Mok CC, Tang SSK, To CH, Petri M. Incidence and risk factors of thromboembolism in systemic lupus erythematosus: a comparison of three ethnic groups. Arthritis Rheum. 2005;52:2774–82. Available from http://www.ncbi.nlm.nih.gov/pubmed/16142761. 10.1002/art.21224 16142761

[12] Ruffatti A, Del Ross T, Ciprian M, Bertero MT, Sciascia S, Salvatore S, et al. Risk factors for a first thrombotic event in antiphospholipid antibody carriers: a prospective multicentre follow-up study. Ann Rheum Dis. 2011;70:1083–6. Available from http://www.ncbi.nlm.nih.gov/pubmed/21285115. 10.1136/ard.2010.142042 21285115

[13] Pengo V, Banzato A, Bison E, Bracco A, Denas G, Ruffatti A. What have we learned about antiphospholipid syndrome from patients and antiphospholipid carrier cohorts?. Semin Thromb Hemost. 2012;38:322–7. Available from http://www.ncbi.nlm.nih.gov/pubmed/22399307. 10.1055/s-0032-1304719 22399307

[14] Pengo V, Ruffatti A, Legnani C, Testa S, Fierro T, Marongiu F, et al. Incidence of a first thromboembolic event in asymptomatic carriers of high-risk antiphospholipid antibody profile: a multicenter prospective study. Blood. 2011;118:4714–8. Available from http://www.ncbi.nlm.nih.gov/pubmed/21765019. 10.1182/blood-2011-03-340232 21765019

[15] Sciascia S, Murru V, Sanna G, Roccatello D, Khamashta MA, Bertolaccini ML. Clinical accuracy for diagnosis of antiphospholipid syndrome in systemic lupus erythematosus: evaluation of 23 possible combinations of antiphospholipid antibody specificities. J Thromb Haemost. 2012;10:2512–8. Available from http://www.ncbi.nlm.nih.gov/pubmed/23025466. 10.1111/jth.12014 23025466

[16] Sciascia S, Bertolaccini ML, Roccatello D, Khamashta MA. Independent validation of the antiphospholipid score for the diagnosis of antiphospholipid syndrome. Ann Rheum Dis. 2013;72:142–3. Available from http://www.ncbi.nlm.nih.gov/pubmed/22843492. 10.1136/annrheumdis-2012-201985 22843492

[17] Sciascia S, Cosseddu D, Montaruli B, Kuzenko A, Bertero MT. Risk Scale for the diagnosis of antiphospholipid syndrome. Ann Rheum Dis. 2011;70:1517–8. Available from http://www.ncbi.nlm.nih.gov/pubmed/21402561. 10.1136/ard.2010.145177 21402561

[18] Cervera R, Serrano R, Pons-Estel GJ, Ceberio-Hualde L, Shoenfeld Y, de Ramón E, et al. Morbidity and mortality in the antiphospholipid syndrome during a 10-year period: a multicentre prospective study of 1000 patients. Ann Rheum Dis. 2015;74:1011–8. Available from http://www.ncbi.nlm.nih.gov/pubmed/24464962. 10.1136/annrheumdis-2013-204838 24464962

[19] Pengo V, Tripodi A, Reber G, Rand JH, Ortel TL, Galli M et al. Update of the guidelines for lupus anticoagulant detection. J Thromb. Haemost. 2009;7:1737–40. Available from http://www.ncbi.nlm.nih.gov/pubmed/19624461. 10.1111/j.1538-7836.2009.03555.x 19624461

[20] Bertolaccini ML, Amengual O, Andreoli L, Atsumi T, Chighizola CB, Forastiero R et al. 14th international congress on antiphospholipid antibodies task force. Report on antiphospholipid syndrome laboratory diagnostics and trends. Autoimmun Rev. 2014;13:917–30. Available from http://www.ncbi.nlm.nih.gov/pubmed/24824074. 10.1016/j.autrev.2014.05.001 24824074

[21] Moore GW, Culhane AP, Daw CR, Noronha CP, Kumano O. Mixing test specific cut-off is more sensitive at detecting lupus anticoagulants than index of circulating anticoagulant. Thromb Res. 2016;139:98–101. Available from http://www.ncbi.nlm.nih.gov/pubmed/26916303. 10.1016/j.thromres.2016.01.019 26916303

[22] Pengo V, Ruffatti A, Tonello M, Hoxha A, Bison E, Denas G, et al. Antibodies to Domain 4/5 (Dm4/5) of β2-Glycoprotein 1 (β2GP1) in different antiphospholipid (aPL) antibody profiles. Thromb Res. 2015;136:161–3. Available from http://www.ncbi.nlm.nih.gov/pubmed/25959581. 10.1016/j.thromres.2015.04.031 25959581

[23] Pengo V, Ruffatti A, Tonello M, Cuffaro S, Banzato A, Bison E, et al. Antiphospholipid syndrome: antibodies to Domain 1 of β2-glycoprotein 1 correctly classify patients at risk. J Thromb Haemost. 2015;13:782–7. Available from http://www.ncbi.nlm.nih.gov/pubmed/25645395. 10.1111/jth.12865 25645395

[24] Meroni PL, Chighizola CB, Rovelli F, Gerosa M. Antiphospholipid syndrome in 2014: more clinical manifestations, novel pathogenic players and emerging biomarkers. Arthritis Res Ther. 2014;16:209. Available from http://www.ncbi.nlm.nih.gov/pubmed/25166960. 10.1186/ar4549 PMC4060447 25166960

[25] Meijide H, Sciascia S, Sanna G, Khamashta MA, Bertolaccini ML. The clinical relevance of IgA anticardiolipin and IgA anti-β2 glycoprotein I antiphospholipid antibodies: a systematic review. Autoimmun Rev. 2013;12:421–5. Available from http://www.ncbi.nlm.nih.gov/pubmed/22951216. 10.1016/j.autrev.2012.08.002 22951216

[26] Bertolaccini ML, Sciascia S, Murru V, Garcia-Fernandez C, Sanna G, Khamashta MA. Prevalence of antibodies to prothrombin in solid phase (aPT) and to phosphatidylserine-prothrombin complex (aPS/PT) in patients with and without lupus anticoagulant. Thromb Haemost. 2013;109:207–13. Available from http://www.ncbi.nlm.nih.gov/pubmed/23254928. 10.1160/TH12-07-0527 23254928

[27] Sciascia S, Khamashta MA, Bertolaccini ML. New tests to detect antiphospholipid antibodies: antiprothrombin (aPT) and anti-phosphatidylserine/prothrombin (aPS/PT) antibodies. Curr Rheumatol Rep. 2014;16:415. Available from http://www.ncbi.nlm.nih.gov/pubmed/24609824. 10.1007/s11926-014-0415-x 24609824

[28] Staub HL, Bertolaccini ML, Khamashta MA. Anti-phosphatidylethanolamine antibody, thromboembolic events and the antiphospholipid syndrome. Autoimmun Rev. 2012;12:230–4. Available from http://www.ncbi.nlm.nih.gov/pubmed/22796282. 10.1016/j.autrev.2012.07.008 22796282

[29] Hirmerova J, Ulcova-Gallova Z, Seidlerova J, Filipovsky J, Bibkova K, Micanova Z, et al. Laboratory evaluation of antiphospholipid antibodies in patients with venous thromboembolism. Clin Appl Thromb Hemost. 2010;16:318–25. Available from http://www.ncbi.nlm.nih.gov/pubmed/19221100. 10.1177/1076029608331228 19221100

[30] Sanmarco M, Gayet S, Alessi M-C, Audrain M, de Maistre E, Gris J-C, et al. Antiphosphatidylethanolamine antibodies are associated with an increased odds ratio for thrombosis. A multicenter study with the participation of the European Forum on antiphospholipid antibodies. Thromb Haemost. 2007;97:949–54. Available from http://www.ncbi.nlm.nih.gov/pubmed/17549297. 17549297

[31] Sanmarco M, Alessi MC, Harle JR, Sapin C, Aillaud MF, Gentile S, et al. Antibodies to phosphatidylethanolamine as the only antiphospholipid antibodies found in patients with unexplained thromboses. Thromb Haemost. 2001;85:800–5. Available from http://www.ncbi.nlm.nih.gov/pubmed/11372671. 11372671

[32] Bertolaccini ML, Roch B, Amengual O, Atsumi T, Khamashta MA, Hughes GR. Multiple antiphospholipid tests do not increase the diagnostic yield in antiphospholipid syndrome. Br J Rheumatol. 1998;37:1229–32. Available from http://www.ncbi.nlm.nih.gov/pubmed/9851275. 10.1093/rheumatology/37.11.1229 9851275

[33] Sanmarco M, Boffa M-C. Antiphosphatidylethanolamine antibodies and the antiphospholipid syndrome. Lupus. 2009;18:920–3. Available from http://www.ncbi.nlm.nih.gov/pubmed/19671793. 10.1177/0961203309106920 19671793

[34] Staub HL, Harris EN, Khamashta MA, Savidge G, Chahade WH, Hughes GR. Antibody to phosphatidylethanolamine in a patient with lupus anticoagulant and thrombosis. Ann Rheum Dis. 1989;48:166–9. Available from http://www.ncbi.nlm.nih.gov/pubmed/2494957. 10.1136/ard.48.2.166 PMC1003707 2494957

[35] Rodriguez-Garcia JL, Bertolaccini ML, Cuadrado MJ, Sanna G, Ateka-Barrutia O, Khamashta MA. Clinical manifestations of antiphospholipid syndrome (APS) with and without antiphospholipid antibodies (the so-called “seronegative APS”). Ann Rheum Dis. 2012;71:242–4. Available from http://www.ncbi.nlm.nih.gov/pubmed/21953349. 10.1136/annrheumdis-2011-200614 21953349

[36] Blaschek MA, Boehme M, Jouquan J, Simitzis AM, Fifas S, Le Goff P, et al. Relation of antivimentin antibodies to anticardiolipin antibodies in systemic lupus erythematosus. Ann Rheum Dis. 1988;47:708–16. Available from http://www.ncbi.nlm.nih.gov/pubmed/3052321. 10.1136/ard.47.9.708 PMC1003586 3052321

[37] Podor TJ, Singh D, Chindemi P, Foulon DM, McKelvie R, Weitz JI, et al. Vimentin exposed on activated platelets and platelet microparticles localizes vitronectin and plasminogen activator inhibitor complexes on their surface. J Biol Chem. 2002;277:7529–39. Available from http://www.ncbi.nlm.nih.gov/pubmed/11744725. 10.1074/jbc.M109675200 11744725

[38] Ortona E, Capozzi A, Colasanti T, Conti F, Alessandri C, Longo A, et al. Vimentin/cardiolipin complex as a new antigenic target of the antiphospholipid syndrome. Blood. 2010;116:2960–7. Available from http://www.ncbi.nlm.nih.gov/pubmed/20634382. 10.1182/blood-2010-04-279208 20634382

[39] Alessandri C, Conti F, Pendolino M, Mancini R, Valesini G. New autoantigens in the antiphospholipid syndrome. Autoimmun Rev. 2011;10:609–16. Available from http://www.ncbi.nlm.nih.gov/pubmed/21545849. 10.1016/j.autrev.2011.04.011 21545849

[40] Arnold J, Holmes Z, Pickering W, Farmer C, Regan L, Cohen H. Anti-beta 2 glycoprotein 1 and anti-annexin V antibodies in women with recurrent miscarriage. Br. J. Haematol. 2001;113:911–4. Available from http://www.ncbi.nlm.nih.gov/pubmed/11442483. 10.1046/j.1365-2141.2001.02812.x 11442483

[41] Nojima J, Kuratsune H, Suehisa E, Futsukaichi Y, Yamanishi H, Machii T, et al. Association between the prevalence of antibodies to beta(2)-glycoprotein I, prothrombin, protein C, protein S, and annexin V in patients with systemic lupus erythematosus and thrombotic and thrombocytopenic complications. Clin Chem. 2001;47:1008–15. Available from http://www.ncbi.nlm.nih.gov/pubmed/11375285. 11375285

[42] Gris JC, Quéré I, Sanmarco M, Boutiere B, Mercier E, Amiral J, et al. Antiphospholipid and antiprotein syndromes in non-thrombotic, non-autoimmune women with unexplained recurrent primary early foetal loss. The Nîmes obstetricians and haematologists study–NOHA. Thromb Haemost. 2000;84:228–36. Available from http://www.ncbi.nlm.nih.gov/pubmed/10959694. 10959694

[43] de Laat B, Derksen RHWM, Mackie IJ, Roest M, Schoormans S, Woodhams BJ, et al. Annexin A5 polymorphism (-1C–&gt;T) and the presence of anti-annexin A5 antibodies in the antiphospholipid syndrome. Ann Rheum Dis. 2006;65:1468–72. Available from http://www.ncbi.nlm.nih.gov/pubmed/16449315. 10.1136/ard.2005.045237 PMC1798354 16449315

[44] Hajjar KA, Jacovina AT, Chacko J. An endothelial cell receptor for plasminogen/tissue plasminogen activator. I. Identity with annexin II. J Biol Chem. 1994;269:21191–7. Available from http://www.ncbi.nlm.nih.gov/pubmed/8063740. 8063740

[45] Kaczan-Bourgois D, Salles JP, Hullin F, Fauvel J, Moisand A, Duga-Neulat I, et al. Increased content of annexin II (p36) and p11 in human placenta brush-border membrane vesicles during syncytiotrophoblast maturation and differentiation. Placenta. 1996;17:669–76. Available from http://www.ncbi.nlm.nih.gov/pubmed/8916217. 10.1016/s0143-4004(96)80017-8 8916217

[46] Cesarman-Maus G, Ríos-Luna NP, Deora AB, Huang B, Villa R, Cravioto M del C, et al. Autoantibodies against the fibrinolytic receptor, annexin 2, in antiphospholipid syndrome. Blood. 2006;107:4375–82. Available from http://www.ncbi.nlm.nih.gov/pubmed/16493010. 10.1182/blood-2005-07-2636 PMC1895790 16493010

[47] Baglin T. The measurement and application of thrombin generation. Br J Haematol. 2005;130:653–61. Available from http://www.ncbi.nlm.nih.gov/pubmed/16115120. 10.1111/j.1365-2141.2005.05612.x 16115120

[48] van Veen JJ, Gatt A, Makris M. Thrombin generation testing in routine clinical practice: are we there yet? Br J Haematol. 2008;142:889–903. Available from http://www.ncbi.nlm.nih.gov/pubmed/18564356. 10.1111/j.1365-2141.2008.07267.x 18564356

[49] Chandler WL, Roshal M. Optimization of plasma fluorogenic thrombin-generation assays. Am J Clin Pathol. 2009;132:169–79. Available from http://www.ncbi.nlm.nih.gov/pubmed/19605810. 10.1309/AJCP6AY4HTRAAJFQ 19605810

[50] Dienava-Verdoold I, Boon-Spijker MG, de Groot PG, Brinkman HJM, Voorberg J, Mertens K, et al. Patient-derived monoclonal antibodies directed towards beta2 glycoprotein-1 display lupus anticoagulant activity. J Thromb Haemost. 2011;9:738–47. Available from http://www.ncbi.nlm.nih.gov/pubmed/21255251. 10.1111/j.1538-7836.2011.04212.x 21255251

[51] Regnault V, Béguin S, Wahl D, de Maistre E, Coenraad Hemker H, Lecompte T. Thrombinography shows acquired resistance to activated protein C in patients with lupus anticoagulants. Thromb Haemost. 2003;89:208–12. Available from http://www.ncbi.nlm.nih.gov/pubmed/12574797. 12574797

[52] Efthymiou M, Lawrie AS, Mackie I, Arachchillage D, Lane PJ, Machin S, et al. Thrombin generation and factor X assays for the assessment of warfarin anticoagulation in thrombotic antiphospholipid syndrome. Thromb Res. 2015;135:1191–7. Available from http://www.ncbi.nlm.nih.gov/pubmed/25895847. 10.1016/j.thromres.2015.03.030 25895847

[53] Zuily S, Ait Aissa K, Membre A, Regnault V, Lecompte T, Wahl D. Thrombin generation in antiphospholipid syndrome. Lupus. 2012;21:758–60. Available from http://www.ncbi.nlm.nih.gov/pubmed/22635224. 10.1177/0961203312440059 22635224

[54] Galli M, Luciani D, Bertolini G, Barbui T. Lupus anticoagulants are stronger risk factors for thrombosis than anticardiolipin antibodies in the antiphospholipid syndrome: a systematic review of the literature. Blood. 2003;101:1827–32. Available from http://www.ncbi.nlm.nih.gov/pubmed/12393574. 10.1182/blood-2002-02-0441 12393574

[55] Pengo V, Ruffatti A, Legnani C, Gresele P, Barcellona D, Erba N et al. Clinical course of high-risk patients diagnosed with antiphospholipid syndrome. J Thromb Haemost. 2010;8:237–42. Available from http://www.ncbi.nlm.nih.gov/pubmed/19874470. 10.1111/j.1538-7836.2009.03674.x 19874470

[56] Sciascia S, Cuadrado MJ, Sanna G, Murru V, Roccatello D, Khamashta MA et al. Thrombotic risk assessment in systemic lupus erythematosus: validation of the global antiphospholipid syndrome score in a prospective cohort. Arthritis Care Res. 2014;66:1915–20. Available from http://www.ncbi.nlm.nih.gov/pubmed/24964745. 10.1002/acr.22388 24964745

[57] Oku K, Amengual O, Bohgaki T, Horita T, Yasuda S, Atsumi T. An independent validation of the global anti-phospholipid syndrome score in a Japanese cohort of patients with autoimmune diseases. Lupus. 2015;24:774–5. Available from http://www.ncbi.nlm.nih.gov/pubmed/25432782. 10.1177/0961203314561284 25432782

[58] Sciascia S, Sanna G, Khamashta MA, Cuadrado MJ, Erkan D, Andreoli L, et al. The estimated frequency of antiphospholipid antibodies in young adults with cerebrovascular events: a systematic review. Ann Rheum Dis. 2015;74:2028–33. Available from http://www.ncbi.nlm.nih.gov/pubmed/24942381. 10.1136/annrheumdis-2014-205663 24942381

[59] Erkan D, Lockshin MD, APS ACTION members. APS ACTION–antiphospholipid syndrome alliance for clinical trials and international networking. Lupus. 2012;21:695–8. Available from http://www.ncbi.nlm.nih.gov/pubmed/22635205. 10.1177/0961203312437810 PMC5733706 22635205

[60] Andreoli L, Chighizola CB, Banzato A, Pons-Estel GJ, Ramire de Jesus G, Erkan D. Estimated frequency of antiphospholipid antibodies in patients with pregnancy morbidity, stroke, myocardial infarction, and deep vein thrombosis: a critical review of the literature. Arthritis Care Res. 2013;65:1869–73. Available from http://www.ncbi.nlm.nih.gov/pubmed/23861221. 10.1002/acr.22066 23861221

[61] Chighizola CB, Andreoli L, de Jesus GR, Banzato A, Pons-Estel GJ, Erkan D, et al. The association between antiphospholipid antibodies and pregnancy morbidity, stroke, myocardial infarction, and deep vein thrombosis: a critical review of the literature. Lupus. 2015;24:980–4. Available from http://www.ncbi.nlm.nih.gov/pubmed/25697769. 10.1177/0961203315572714 25697769

